# Supplementary material for: In Silico and In Vivo Studies of a Tumor-Penetrating and Interfering Peptide with Antitumoral Effect on Xenograft Models of Breast Cancer
Source: Pharmaceutics. 2023 Apr 7;15(4):1180. doi: 10.3390/pharmaceutics15041180 (PMC10142558; doi:10.3390/pharmaceutics15041180)
Supplement: Supplementary file 1 [file pharmaceutics-15-01180-s001.zip › pharmaceutics-2197144-supplementary.pdf]

SUPPLEMENTARY INFORMATION

FACING *IN SILICO* RESULTS OF A TUMOR PENETRATING AND INTERFERING PEPTIDE WITH ANTITUMORAL EFFECT ON XENOGRAFT MODELS OF BREAST CANCER

Gustavo H. Marin[1†], Samuel Murail[2†], Laura Andrini[1], Marcela Garcia[1], Severine Loisel[3], Pierre Tuffery[2‡] and Angelita Rebollo[4*‡]

1 Department of Pharmacology/Histology and Embryology, FMC, National University of La Plata, CONICET, Argentina

2 BFA, Université Paris Cite, CNRS UMR 8251, Inserm U1133, Paris, France

3 Animalerie de l'UBO, UFR Médicine, Brest, France

4 UTCBS, Université Paris Cite, Inserm U1267, Paris, Faculté de Pharmacie, France

***Correspondence to : Angelita Rebollo, UTCBS, Université Paris Cite, Faculté de Pharmacie, 4 avenue de l'observatoire, 75006 Paris, France.

E-mail: angelita.rebollo@parisdescartes.fr

† The two first authors equally contributed to this work

‡ The two last authors equally contributed to this work

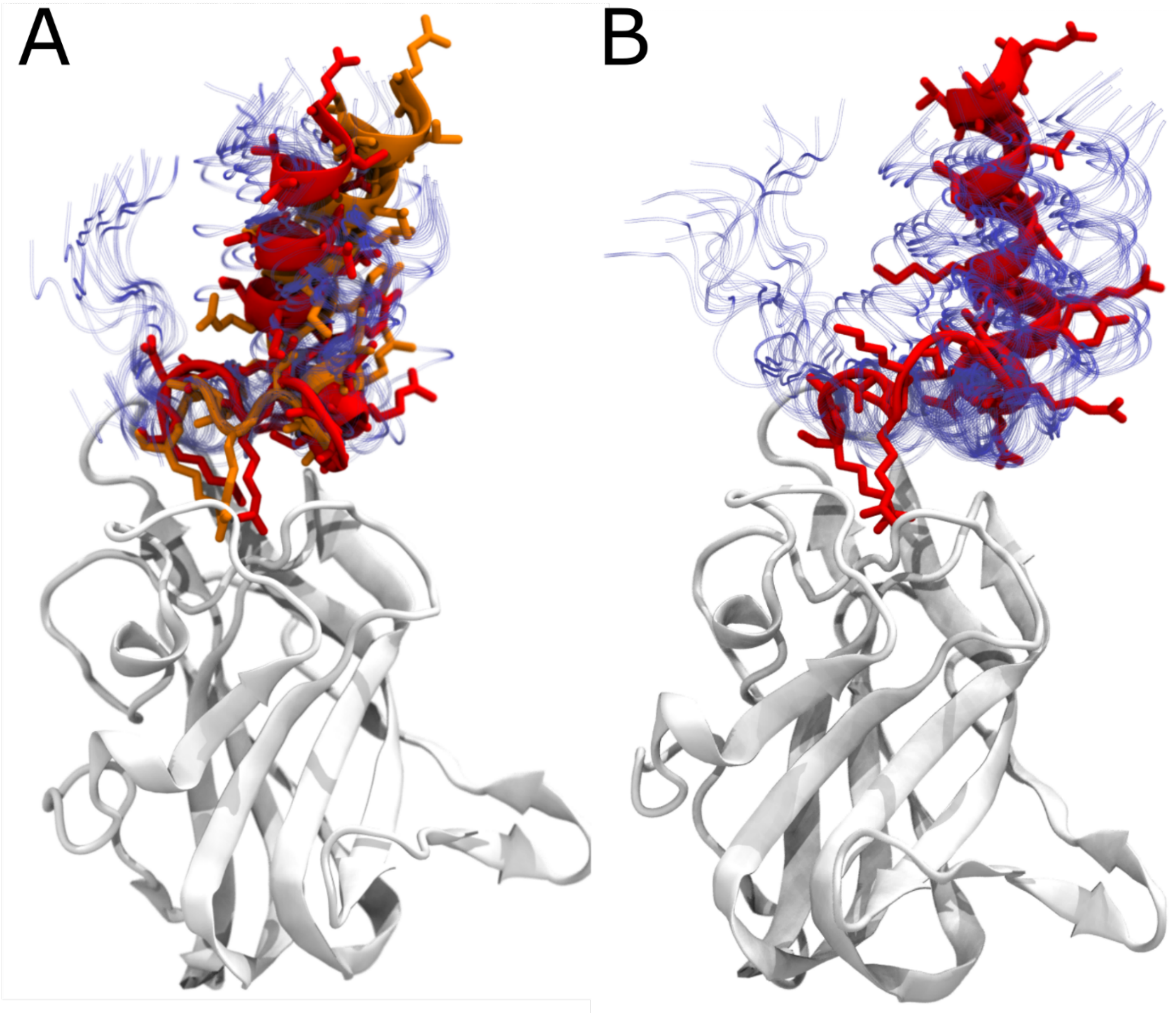

Figure S1. Superimposed alphafold models of IP-GG-iRGD-cl **(A)** and IP-GG-LinTT1-cl **(B).** Neuropilin1 is shown as a white cartoon, the 50 peptide models are shown as blue transparent ribbons and the best peptides were shown in colored cartoons according to scatterplot colors (orange and red) in Figure 1, for the same models the peptide atoms were shown as colored sticks.

IP-GG-LinTT1-cl

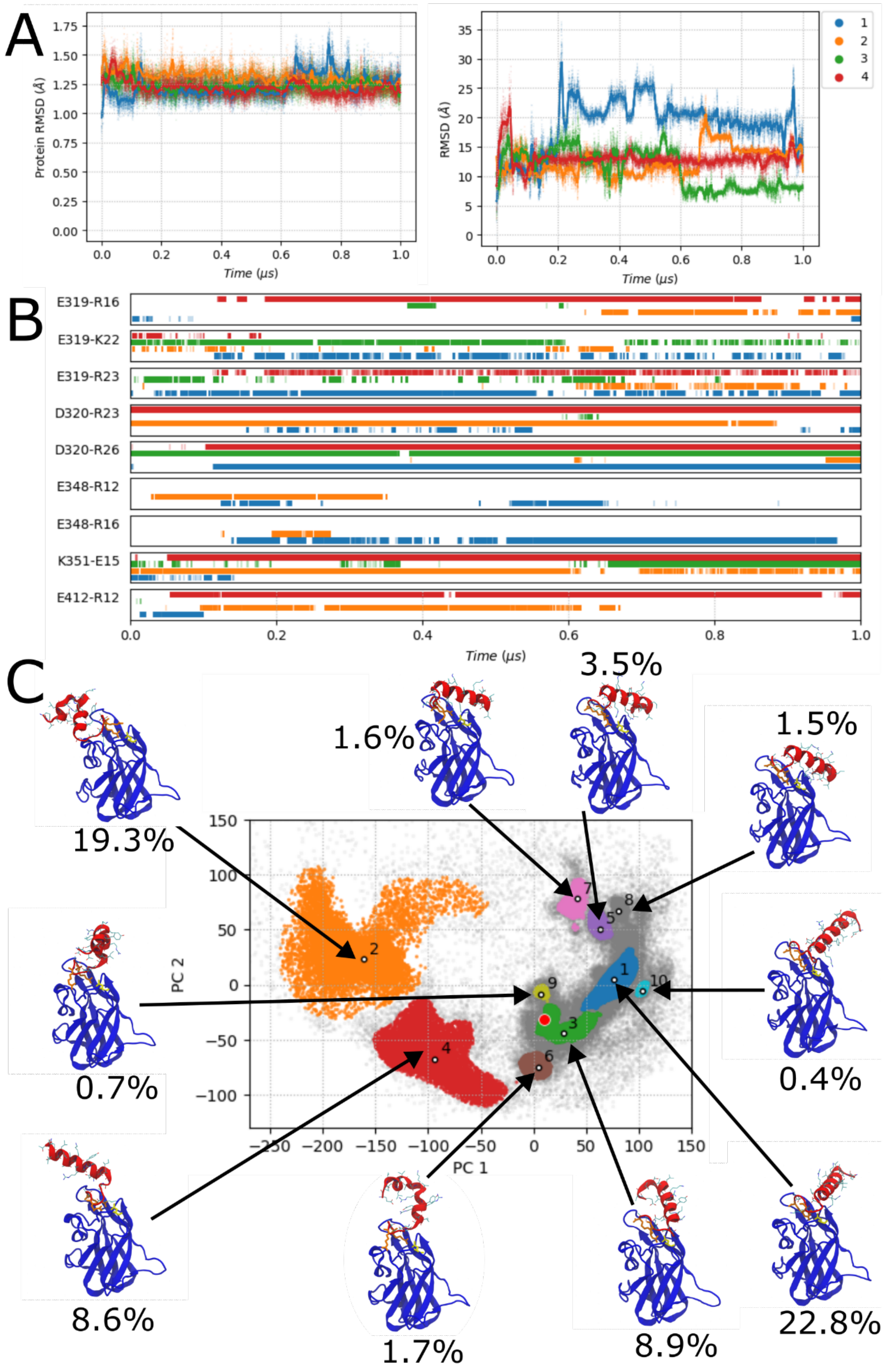

Figure S2. Dynamic of IP-GG-LinTT1-cl peptide and its interaction with Neuropilin-1. **(A)** The left panel displays Neuropilin-1 backbone atoms RMSD to the initial alphafold model colored by simulation replicas, the right panel displays RMSD of peptide backbone atoms to the initial alphafold model after structure fitting on the Neuropilin-1 backbone atoms, colored by simulation replicas. **(B)** Detail of salt bridges between peptide IP-GG-LinTT1-cl and Neuropilin-1, each plot represents a salt bridge labeled on the left as a bar code plot colored by replicas. A salt bridge is considered if the distance between, on one side, Arginine C_ζ atoms and Lyzine N_ζ atoms and on the other side, Aspartic acid C_γ and Glutamic acid C_δ atoms was below 5.0 Å. **(C)** Structural landscape explored by the peptide backbone during MD simulation. Principal Component Analysis (PCA) was applied to the four MD simulation replicas. Projection of first (64% of variance, *x*-axis) and second (13% of variance *y*-axis) PCA modes computed on peptide backbone atoms after trajectory fitting on receptor backbone atoms is colored according to cluster number. Clusterization was made using the HDBSCAN algorithm[1]. For each cluster, the closest frame in terms of RMSD to the average cluster structure is shown around the plot. Peptide is represented as a red cartoon, as the Neuropilin-1 is depicted as a blue cartoon. The peptide residues R23 and R26 are depicted as orange licorice and Neuropilin-1 D320 residue is shown as yellow licorice.

IP-GG-iRGD-clp

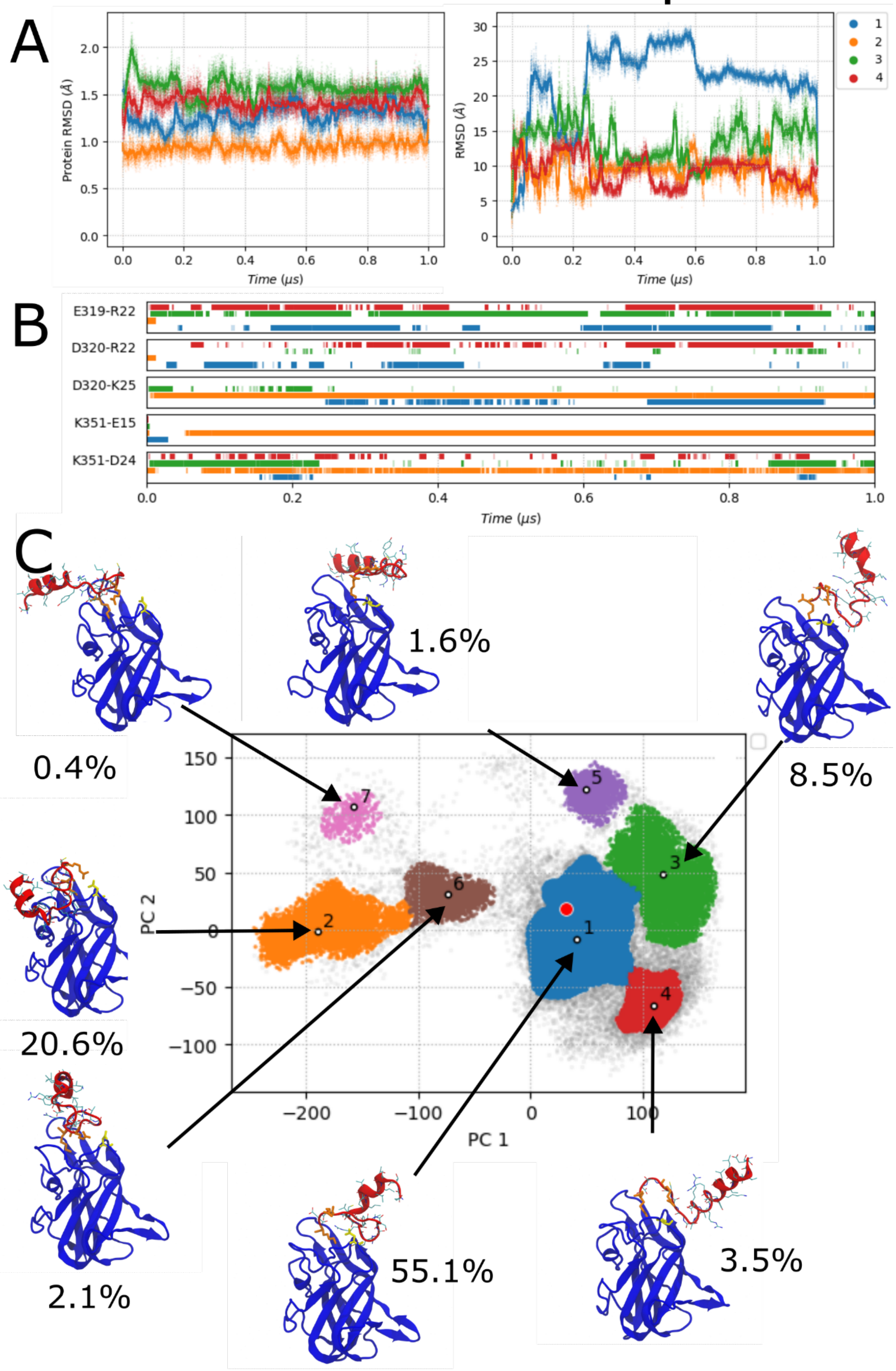

Figure S3. Dynamic of IP-GG-iRGD-clp peptide and its interaction with Neuropilin-1. **(A)** The left panel displays Neuropilin-1 backbone atoms RMSD to the initial alphafold model colored by simulation replicas, the right panel displays RMSD of peptide backbone atoms to the initial alphafold model after structure fitting on the Neuropilin-1 backbone atoms, colored by simulation replicas. **(B)** Detail of salt bridges between peptide IP-GG-LinTT1-cl and Neuropilin-1, each plot represents a salt bridge labeled on the left as a bar code plot colored by replicas. A salt bridge is considered if the distance between, on one side, Arginine C_ζ atoms and Lyzine N_ζ atoms and on the other side, Aspartic acid C_γ and Glutamic acid C_δ atoms was below 5.0 Å. **(C)** Structural landscape explored by the peptide backbone during MD simulation. Principal Component Analysis (PCA) was applied to the four MD simulation replicas. Projection of first (70% of variance, *x*-axis) and second (10% of variance *y*-axis) PCA modes computed on peptide backbone atoms after trajectory fitting on receptor backbone atoms is colored according to cluster number. Clusterization was made using the HDBSCAN algorithm[1]. For each cluster, the closest frame in terms of RMSD to the average cluster structure is shown around the plot. Peptide is represented as a red cartoon, as the Neuropilin-1 is depicted as a blue cartoon. The peptide residues R23 and R26 are depicted as orange licorice and Neuropilin-1 D320 residue is shown as yellow licorice.

References

1. Campello, R.J.G.B.; Moulavi, D.; Sander, J. Density-Based Clustering Based on Hierarchical Density Estimates. In Proceedings of the Advances in Knowledge Discovery and Data Mining; Pei, J., Tseng, V.S., Cao, L., Motoda, H., Xu, G., Eds.; Springer: Berlin, Heidelberg, 2013; pp. 160–172.
